# The prognostic value of circulating tumour cells (CTCs) and CTC white blood cell clusters in patients with renal cell carcinoma

**DOI:** 10.1186/s12885-021-08463-7

**Published:** 2021-07-17

**Authors:** Yibing Guan, Fangshi Xu, Juanhua Tian, Ke Gao, Ziyan Wan, Yiyuan Wang, Mei Gao, Zhenlong Wang, Tie Chong

**Affiliations:** 1 Department of Urology, the Second Affiliated Hospital, School of Medicine, Xi’an Jiaotong University, No 157 Xiwu Road, Xi’an, 710004 Shaan Xi Province China; 2 School of Medicine, Xi’an Jiaotong University, Xi’an, China; 3 Department of Stomatology, the Second Affiliated Hospital of Shaanxi University of Traditional Chinese Medicine, Xianyang, China

**Keywords:** Renal cell carcinoma, CTC-WBC cluster, Circulating tumour cells, Metastasis-free survival, Prognosis

## Abstract

**Purpose:**

Circulating tumour cell (CTC) and CTC-white blood cell (CTC-WBC) clusters are related to the prognosis of tumour patients. However, the relationship between CTC-WBC clusters and prognosis in renal cell carcinoma (RCC) patients is not clear. We evaluated the prognostic value of CTC-WBC clusters using metastasis-free survival (MFS) and overall survival (OS) in patients with RCC.

**Materials and methods:**

The baseline, survival, and CTC data of patients with RCC were statistically analysed by R.

**Results:**

The Cox risk proportional regression model suggests that the total CTCs, pathology type, and CTC-WBC clusters can be used as prognostic indicators for the MFS of RCC patients. Total CTCs and solid tumour diameter can be used as prognostic indicators for the OS of RCC patients. Using Kaplan–Meier survival analysis, we found that patients with total CTCs, pathology, and CTC-WBC clusters greater than the cut-off value had a worse MFS, and patients with total CTCs greater than the cut-off value had a worse OS.

**Conclusion:**

The analysis of the clinical sample data in patients with RCC shows that CTC-WBC clusters play an important role in monitoring the prognosis of RCC. Among them, total CTCs, pathology, and CTC-WBC clusters were combined as prognostic factors for the MFS of RCC patients. Total CTCs and solid tumour diameter can be combined as prognostic factors for the OS of RCC patients. These prognostic factors provide more convenient and accurate condition monitoring for renal cancer patients and can be used to actively improve the prognosis of patients.

**Supplementary Information:**

The online version contains supplementary material available at 10.1186/s12885-021-08463-7.

## Background

Renal cell carcinoma (RCC) is a common tumour of the urinary system, accounting for 80 to 85% of all kidney tumours and 3.8% of all newly diagnosed tumours [1]. RCC has more than 15 subtypes based on tissue classification, among which clear cell RCC (ccRCC) accounts for ∼75% of these malignant tumours, followed by papillary (types 1 and 2; 15%) and chromophobe subtypes (5%) [2, 3]. Most patients have only local symptoms at the time of diagnosis. However, 30% of patients will have tumour recurrence or metastasis after surgery, and the 5-year survival rate of these patients is only 11% [4, 5]. At present, RCC still lacks specific disease indicators to conduct simple and effective disease monitoring, guide treatment measures, and improve patient prognosis.

Circulating tumour cells (CTCs) are tumour cells shed from solid tumours that enter the circulating blood and are transferred to remote organs. They are considered the seeds of tumour metastasis and recurrence and can be used as various tumour metastasis and prognostic indicators [6]. However, due to the scarcity and heterogeneity of CTCs, their predictive value is limited [7]. In hepatic cell carcinoma (HCC), Chen found that CTC count and epithelial-mesenchymal transition (EMT) classification were not related to clinical staging or prediction of HCC recurrence [8]. Therefore, CTC clusters composed of several CTCs or CTCs and neutrophils have attracted wide attention from researchers because neutrophils act as “hitchhikers” to escort CTCs to the whole body through various mechanisms; for example, neutrophils release neutrophil extracellular traps (NETs) containing nuclear DNA to capture CTCs and promote tumour metastasis [9, 10]. More importantly, neutrophils can interact with CTCs to systematically drive the cell cycle process and expand the metastatic potential of CTCs [11]. In addition, in HCC, the CTC-white blood cell (CTC-WBC) cluster is a poor prognostic factor [12].

In RCC, the prognostic value of CTC-WBC clusters is not clear. Therefore, we evaluated CTCs and CTC-WBC clusters in the peripheral blood of 163 RCC patients and explored the value of the CTC-WBC cluster in predicting the metastasis-free survival (MFS) and overall survival (OS) to clarify the clinical effects of CTC-WBC clusters on disease monitoring.

## Method

### Patient clinical data

With the approval of the ethics review committee of the Second Affiliated Hospital of Xi’an Jiaotong University, we included a total of 163 RCC patients who had undergone surgery in our hospital between September 2015 and January 2019. The institutional review boards approved the study using clinical samples in the Second Affiliated Hospital of Xi’an Jiaotong University, and written informed consent was obtained from all participants. All methods were performed following the Declaration of Helsinki. The clinical data of the patients included sex, age, tumour stage, pathological type, etc. CTC data collected included counts of both CTCs and CTC-WBC clusters. Data regarding the progress of patients was obtained from the database of our hospital’s follow-up system. Blood was drawn from patients for CTC testing approximately three months after surgery. None of the patients were treated with steroids or anti-inflammatory drugs that could affect blood immune cell counts before blood sample collection.

### CTCs and CTC-WBC clusters test

According to the operating requirements of CTC testing, peripheral blood samples (5 ml, anticoagulated with EDTA) were drawn from all participants. Mononuclear cells were isolated by adding erythrocyte lysis buffer (Sigma, St. Louis, USA). After centrifugation (1500 rpm, 5 min), the blood samples were resuspended in PBS buffer. CTCs were separated by using the CanPatrol CTC enrichment technique (SurExam, Guangzhou, China). The details of the protocol are described below.

The filter was connected to the vacuum pump through the vacuum manifold. The liquid was transferred from the sample storage tube to the filter. The mononuclear cells in the solution were left on the nanomembrane of the filter. Then, the nanomembrane was removed from the filter and laid on the slide. The cells were fixed with formaldehyde for 60 min at room temperature.

The fixed samples were typed by RNA in situ hybridization to determine the type of CTCs. The permeation agent was incubated for 5 min and washed with PBS three times. Digestive enzymes were incubated for 60 min and washed with PBS three times. The working solution of the probe was added and incubated at 40 ± 1 °C for 3 h and was washed with PBS three times. The preamplification working solution was added and incubated at 40 ± 1 °C for 30 min and was washed with PBS three times. The amplification solution was added and incubated at 40 ± 1 °C for 30 min and was washed with PBS three times. The chromogenic working solution was added and incubated at 40 ± 1 °C for 30 min and was washed with PBS three times. An anti-quenching agent (including DAPI) was added to the sample.

The CTC typing technique used multiple RNA probes to detect the epithelial type-specific genes EpCAM, CK8, CK18, and CK19 and mesenchymal type-specific genes Vimentin and Twist (the probe sequence is shown in Table S1). The amplification probe was hybridized with the above labelling probe labelled with fluorescent groups to produce fluorescence signals. The automatic recognition system read the fluorescence signals, and the results of CTC typing were analysed automatically through different colour fluorescence signals.

CTCs were divided into epithelial, mesenchymal, and hybrid phenotypes based on morphological and biological biomarkers. The epithelial CTCs only showed the epithelial markers (EpCAM and CK8/18/19) marked by Alexa Fluor 594 (red), and the mesenchymal CTCs only showed the mesenchymal markers (Vimentin and Twist) marked by Alexa Fluor 488 (green). Hybrid CTCs with both epithelial and mesenchymal markers were stained with green and red immunofluorescence simultaneously. Nuclei were labelled with DAPI and were visible as a blue fluorescence. CTC-WBC clusters were seen as white dots of WBCs around a red, green, or red/green mixture of CTCs. After labelling, the cells were analysed with a fluorescence microscope.

### Statistical analysis

Statistical analysis was performed in R using t-tests and chi-square tests to analyse the baseline data after the patients were grouped. A univariate Cox risk proportional regression model was used to evaluate the prognostic value of CTCs and other indicators. Best subset selection was used to screen the best combination of indicators from many candidate indicators to build the best model. A multivariate Cox risk proportional regression model was used to verify the prognostic value of the best combination of indicators and to visualize the Cox risk proportional regression model through a nomogram. The C-index and calibration plot were used to evaluate the quality and effectiveness of the multivariate Cox risk proportional regression model. The R maxstat package was used to obtain the best cut-off values of the various indicators within the survival data, and the continuous data was processed into two categories. Kaplan-Meier survival curves were used to evaluate the effects of the indicators as prognostic factors. In general, *P* < 0.05 indicated that the difference was statistically significant.

## Results

### Patient disease progression and baseline data analysis

A total of 163 RCC patients were included in this study, of which 42 (25.8%) had metastases and 8 (4.9%) died. By comparing the metastatic group and the metastatic-free group, it was found that the difference in pathological subtype was statistically significant. The differences in age, sex, T stage, renal score, differentiation, and surgical approach were not statistically significant (Table 1). Through the comparison of the survival group and the death group, it was found that the differences in various aspects were not statistically significant (Table S2).

**Table 1:** Association of disease status with clinical pathological variables

	Status		*p*^*1*^
	Metastasis-free (*n* = 121)	Metastasis (*n* = 42)	
Age	55.7	54.6	0.553^2^
Diameter	5.2	5.5	0.554
Gender			
Male	76	31	0.196
Female	45	11	
Pathological subtype			
Clear cell carcinoma	103	29	0.022
Others	18	13	
T stage (*n* = 160)^3^			
T1	98	32	0.862
T2	17	7	
T3	5	1	
Renal score (*n* = 134)			
Low risk	21	5	0.592
Medium risk	48	20	
High risk	30	10	
Differentiation (*n* = 124)			
Low	19	11	0.305
Middle	4	4	
High	62	24	
Surgical approach			
Radical resection	51	19	0.728
Partial resection	70	23	

^1^Chi- square test

^2^Independent sample T test

^3^Some patients data are missing

### CTCs and CTC-WBC clusters detection

**Fig. 1:**
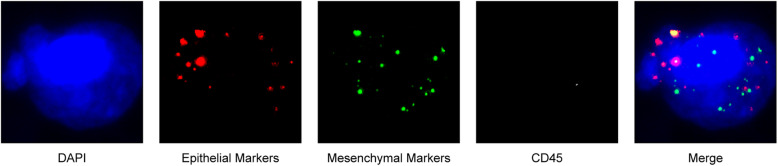
Examples of CTCs, WBCs, and CTC-WBC clusters under automated fluorescence microscopy imaging. Epithelial CTCs stained with EpCAM or CK8/18/19 (red). Mesenchymal CTCs stained with vimentin or Twist (green). WBCs stained with CD45 (white). Nuclei stained with DAPI (blue). DAPI 40,6-diamidino-2-phenylindole

The CanPatrol™ CTC analysis system (SurExam, China) was used to detect the number of CTCs and CTC-WBC clusters in 5 ml of whole samples of peripheral blood. CTC-WBC clusters were seen as white dots of WBCs around a red, green, or red/green mixture of CTCs. After labelling, the cells were analysed with a fluorescence microscope (Fig. 1). The patient’s CTC and CTC-WBC cluster counts ranged from 0 to 104 and 0 to 3, respectively (Fig. S1).

### Cox proportional hazards model

**Fig. 2:**
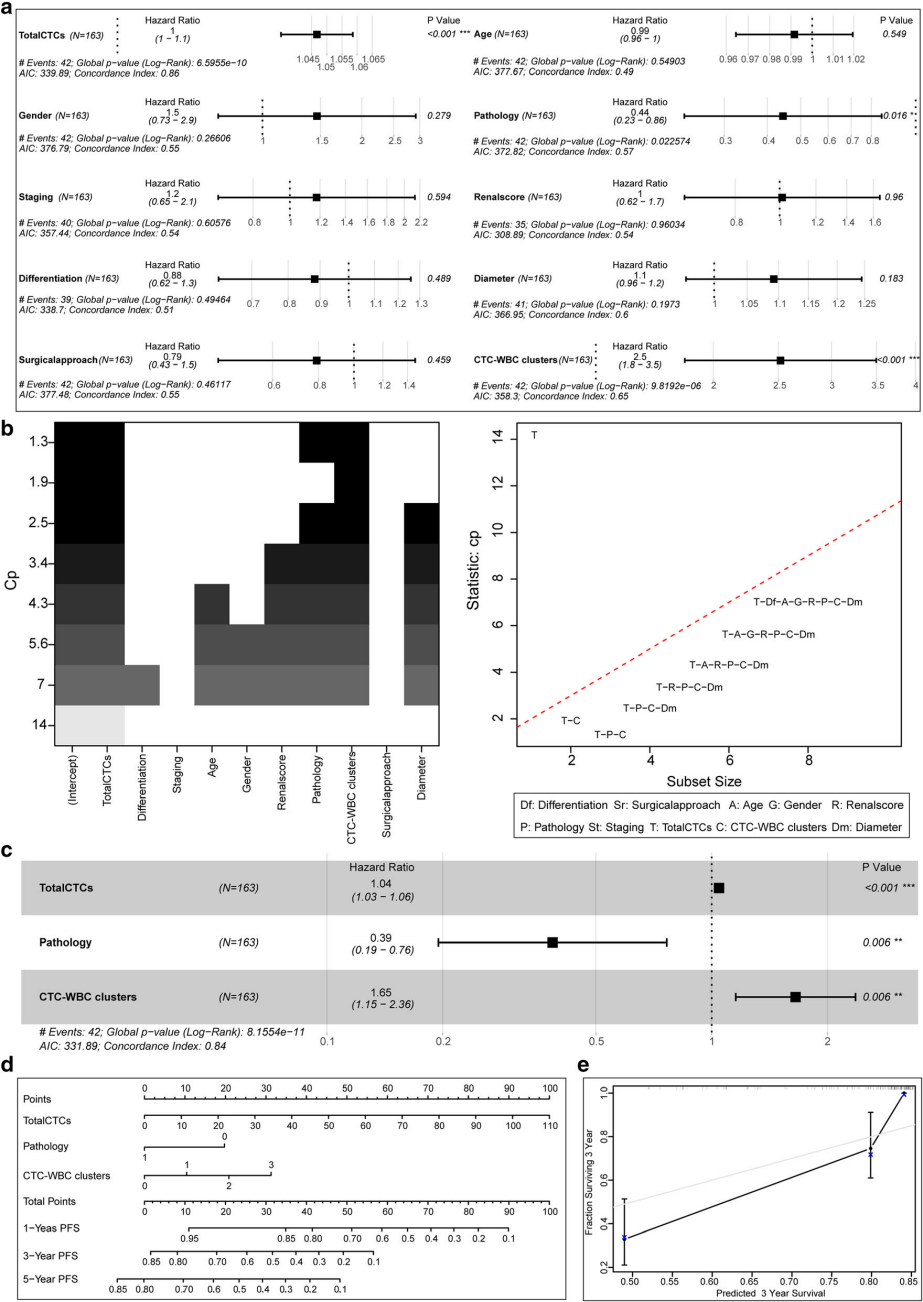
Cox regression model of MFS and its effect verification. **a**: Univariate Cox risk proportional regression model for analysis of various indicators. **b**: The best subset selection function filters the indicators for the multivariate Cox risk proportional regression model. **c**: Multivariate Cox risk proportional regression model on the selected indicators. **d**: Multivariate Cox risk proportional regression model with nomogram visualization. **e**: Calibration plot evaluation of the multivariate Cox risk proportional regression model prediction effect

The univariate Cox risk proportional regression model suggested that in the metastasis-free and metastasis groups, the *P* values of total CTCs, pathology, and CTC-WBC clusters were all less than 0.05, which was statistically significant in the model (Fig. 2A). All factors were evaluated through the best subset selection function. It was confirmed that total CTCs, pathology, and CTC-WBC clusters could be used as components of the optimal model (Fig. 2B). In the multivariate Cox risk proportional regression model, the *p* values of total CTCs, pathology, and CTC-WBC clusters were all less than 0.05 (Fig. 2C), which was statistically significant in the model. At the same time, the nomogram visualized the multivariate Cox risk proportional regression model (Fig. 2D). Model evaluation confirmed that its C-Index of 0.84 has a high accuracy, and the actual curve of the 3-year MFS rate fits well with the calibration curve (Fig. 2E).

**Fig. 3:**
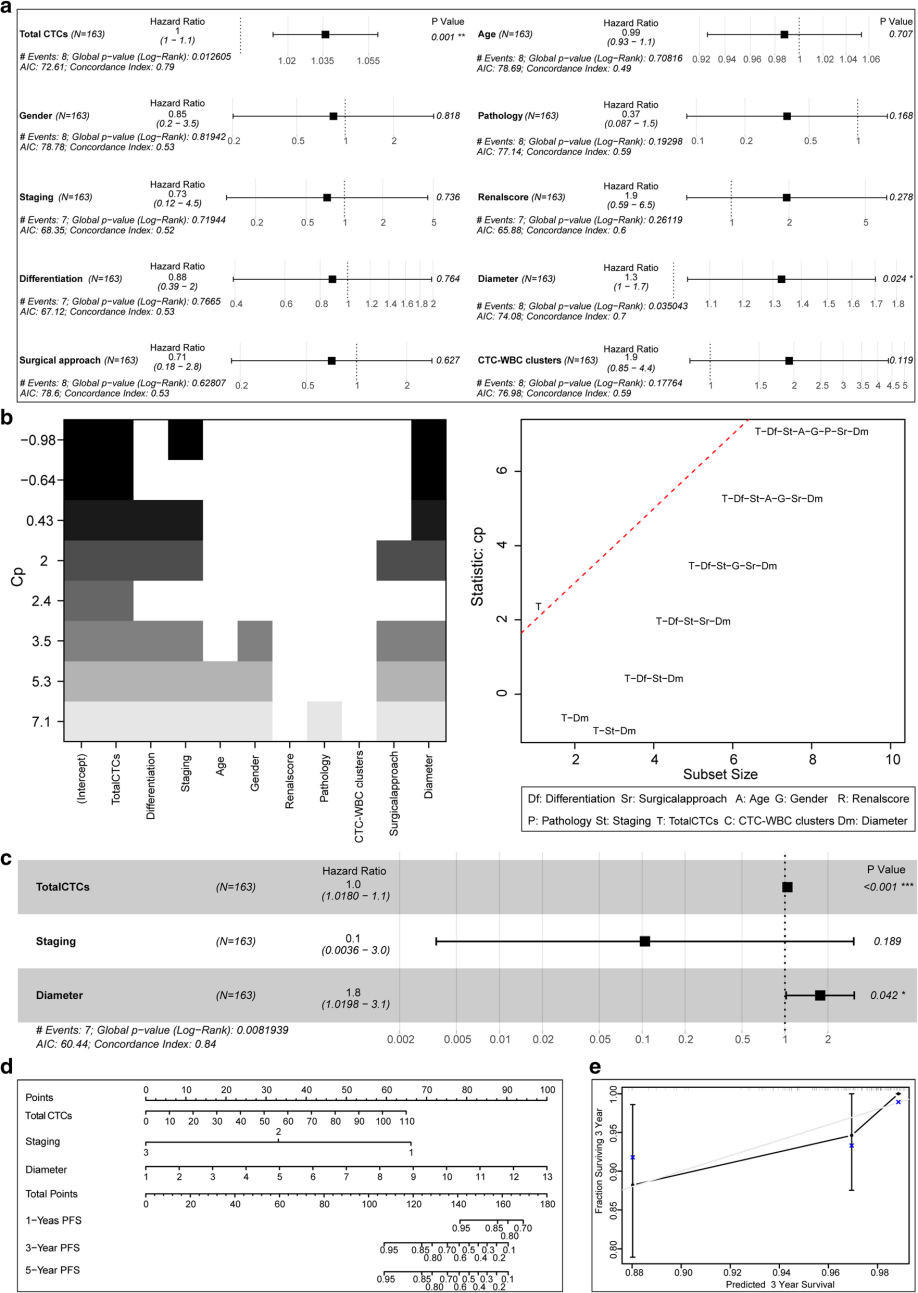
Cox regression model of OS and its effect verification. **a**: Univariate Cox risk proportional regression model for analysis of various indicators. **b**: The best subset selection function filters the indicators for the multivariate Cox risk proportional regression model. **c**: Multivariate Cox risk proportional regression model on the selected indicators. **d**: Multivariate Cox risk proportional regression model with nomogram visualization. **e**: Calibration plot evaluation of the multivariate Cox risk proportional regression model prediction effect

The univariate Cox risk proportional regression model indicated that the *p*-value of total CTCs and solid tumour diameter were less than 0.05 in both the survival group and in the death group, which were statistically significant in the model (Fig. 3A). All factors were evaluated through the best subset selection function. It was confirmed that total CTCs, staging, and solid tumour diameter can be used as the components of the optimal model (Fig. 3B). In the multivariate Cox risk proportional regression model, the *P* values of total CTCs and solid tumour diameter were both less than 0.05 (Fig. 3C), which was statistically significant in the model. At the same time, the nomogram visualized the multivariate Cox risk proportional regression model (Fig. 3D). Model evaluation confirmed that its C-Index of 0.84 has a high accuracy, and the actual curve of the 3-year OS rate fits well with the calibration curve (Fig. 3E).

### Cut-off value and Kaplan-Meier curve analysis

**Fig. 4:**
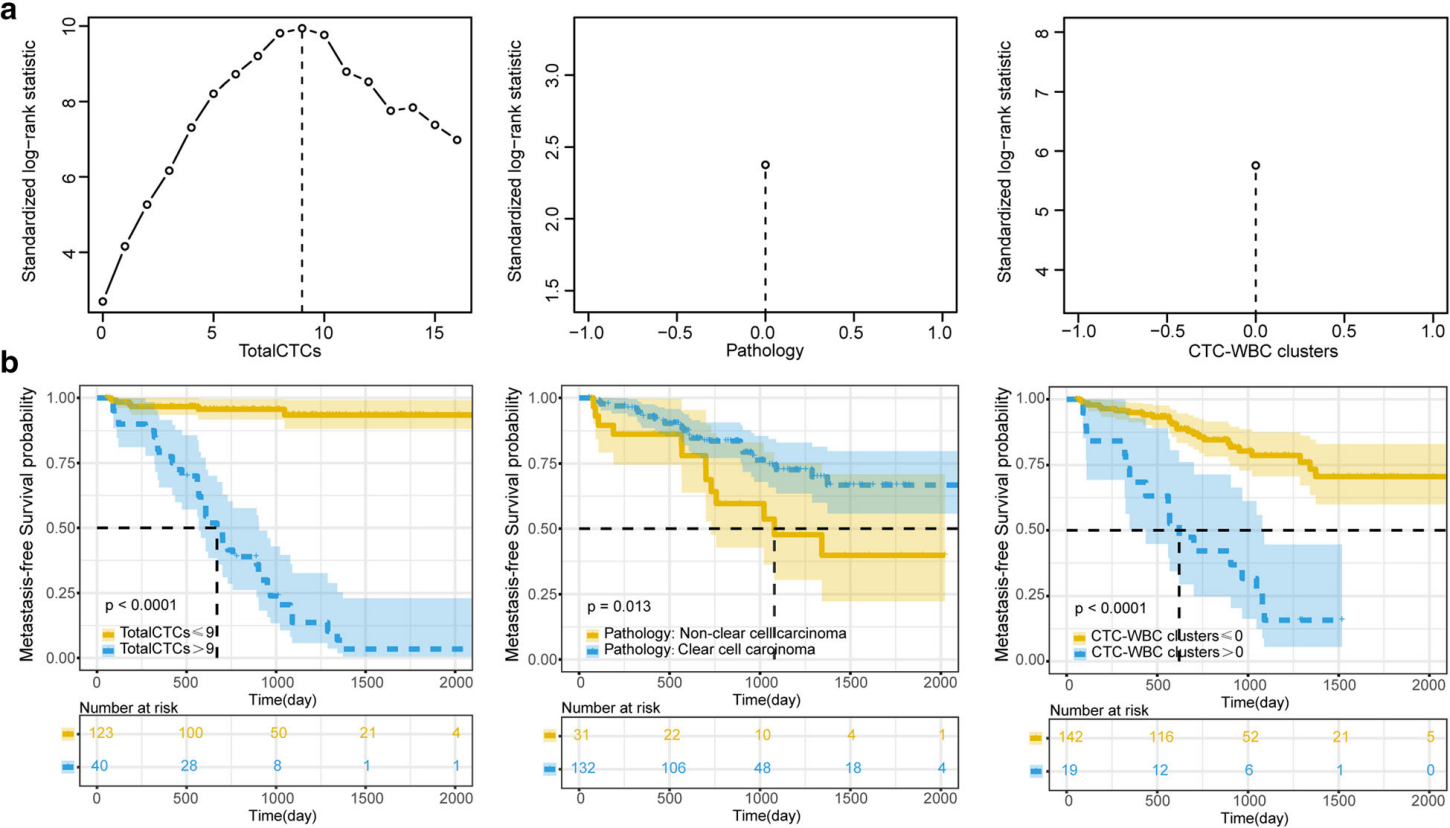
MFS candidate index cut-off value and Kaplan-Meier curve analysis. **a**: The R maxstat package was used to analyse the best cut-off values of the various indicators of the survival data, and the continuous data were processed into two categories. **b**: Kaplan-Meier curve for survival analysis and statistical calculation of candidate binary classification indicators

**Fig. 5:**
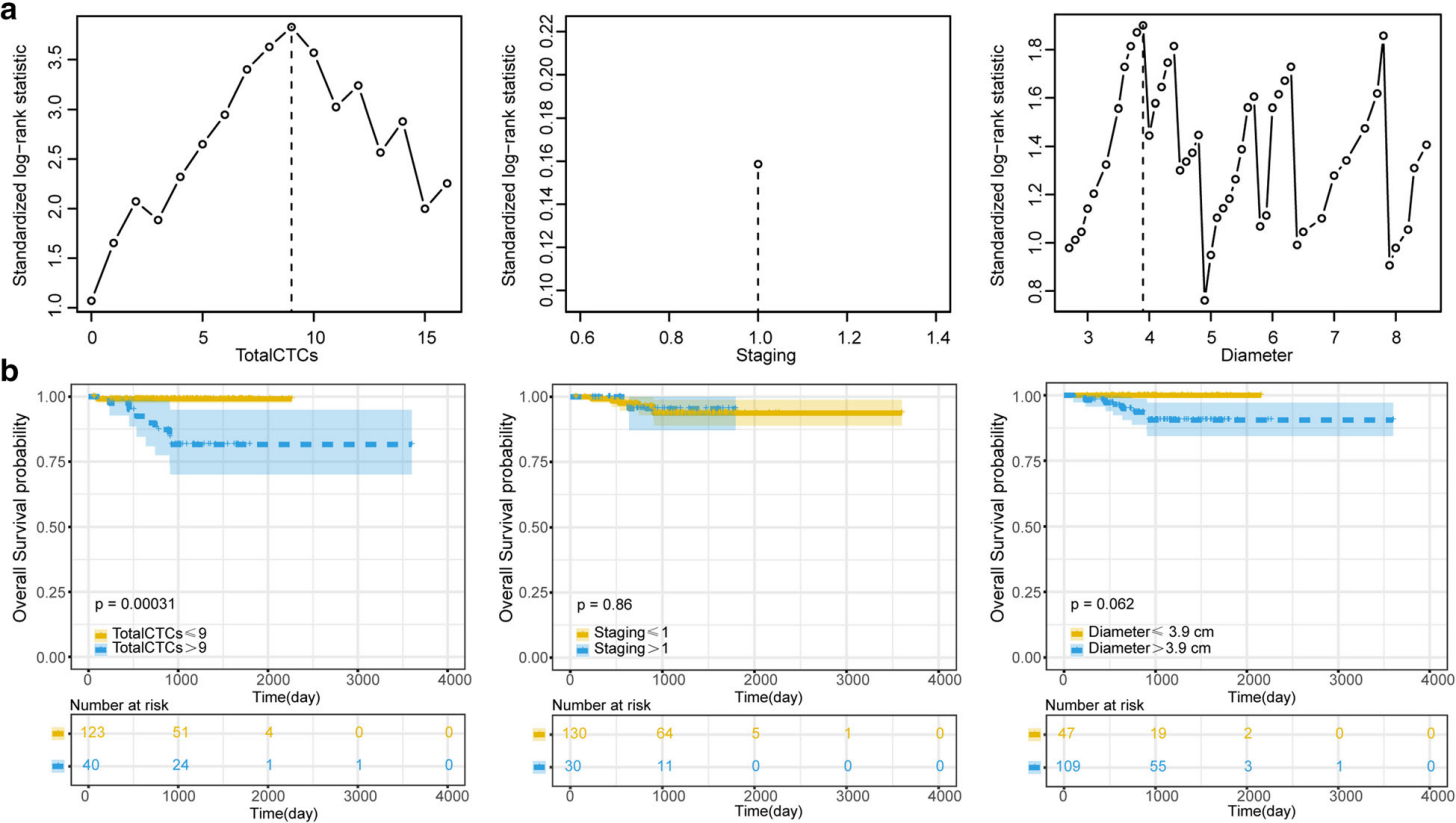
OS candidate index cut-off value and Kaplan-Meier curve analysis. **a**: The R maxstat package was used to analyse the best cut-off values of the various indicators of the survival data, and the continuous data were processed into two categories. **b**: Kaplan-Meier curve for survival analysis and statistical calculation of candidate binary classification indicators

In both the metastatic group and the metastasis-free group, the cut-off values of total CTCs, pathology, and CTC-WBC clusters were 9, 0, and 0, respectively (Fig. 4A). Based on this standard, the total CTCs, pathology, and CTC-WBC clusters were classified into two categories. Kaplan-Meier curves suggested that the *P* values of the total CTCs, pathology and CTC-WBC clusters were all less than 0.05, which was statistically significant. Among them, total CTCs and CTC-WBC clusters were more effective as indicators than pathology (Fig. 4B), suggesting that total CTCs and CTC-WBC clusters can more accurately predict the prognosis of patients. The patient survival data of the metastatic group and the metastatic-free group are shown in Table S3. In the survival and death groups, the cut-off values of total CTCs, staging, and solid tumour diameter were 9, 1, and 3.9, respectively (Fig. 5A). Based on this standard, the total CTCs, staging, and diameter were classified into two categories. Kaplan-Meier curves indicated that the *P* values of total CTCs were all less than 0.05, which was statistically significant. The results of staging and diameter were not statistically significant (Fig. 5B). The patient survival data of the survival group and the death group are shown in Table S4.

## Discussion

CTCs, as tumour cells shed from the primary tumour into the blood circulatory system, play a crucial role in the haematogenous transmission of tumours [13]. As early as 2005, Cristofanilli reported that CTCs could be used as effective predictors of the progression-free survival and OS in breast cancer patients [14].

Some studies have pointed out the difficultly in adequately predicting the prognosis by relying solely on the relevant information of CTCs. For example, the androgen receptor variant 7AR-V7 expression of CTCs had no prognostic value in patients with prostate cancer [15]. CTC count and EMT classification in hepatocellular carcinoma were not related to the clinical stage or prediction of HCC recurrence [8]. Therefore, researchers need to find other relevant supplemental indicators to improve the accuracy of the CTC fluid biopsy. In previous studies, we found that CTCs have an EMT process, and mesenchymal CTCs are closely related to the metastasis or recurrence of RCC patients [16, 17]. However, there are no previous studies reporting the value of clusters with CTCs in the prognosis of RCC. Therefore, in this study, we explored the prognostic value of CTC and CTC clusters in patients with RCC.

CTCs face the threat of the shear forces in blood flow, of anoikis, and of the response of the immune system within the blood [10]. Therefore, the half-life of CTCs is very short, approximately 2 h [18]. In response to the above threats, CTCs can adopt EMT [19], bind with platelets [20], and activate tropomyosin-related kinase B [21]. Among them, the interaction between CTCs and neutrophils is essential [22]. Neutrophils are part of the natural immune system and form the most significant proportion of WBCs in human circulation [12]. In our previous study, the differentially expressed gene functions of CTCs and primary tumour cells were mainly enriched with leukocyte activation adhesion and inflammatory immune response (Fig. S2), suggesting a close relationship between CTCs and neutrophils, especially WBCs [16].

Nevertheless, neutrophils have a double-edged sword effect. On the one hand, when neutrophils are in direct contact with tumour cells, they can produce TNF-α, IL-1β, proteases, membrane perforating agents, and other compounds to eliminate tumour cells; on the other hand, the gastrointestinal tract and other malignant tumours are characterized by neutrophil infiltration, which enhances tumour cell invasiveness [22–24]. Although neutrophils were initially thought to have a defensive function against tumour cells, some neutrophils have been found to have tumour-promoting effects by acting on circulating tumour cells. For instance, Yoshiharu proposed that neutrophils promote tumour cell dissemination by capturing circulating tumour cells using neutrophil extracellular traps and promoting tumour cell migration to distant sites [25]. Aceto proposed a model in which the association between CTCs and neutrophils supports cell cycle progression within the bloodstream and expands the metastatic potential of CTCs [11]. Lorenzo confirmed using animal experiments that circulating tumour cells become trapped within NETs, and NET trapping was associated with the increased formation of hepatic micrometastases at 48 h and an increase in gross metastatic disease burden at 2 weeks following tumour cell injection [26].

In clinical applications, CTCs are often used as a “liquid biopsy” method for tumour screening, patient prognosis, and disease monitoring [6]. The clinical analysis of CTCs has also developed from the initial quantitative typing of CTCs to a comprehensive analysis of CTCs combined with multiple indicators and multiple detection methods [6]. Due to the close relationship between CTCs and peripheral blood leukocytes, such as in the tumour microenvironment, tumour-related neutrophils (TANs) promote the growth and metastasis of cancer cells by directly acting on cancer cells and indirectly affecting tumour cells by changing the tumour microenvironment [27]; therefore, CTC-WBC clusters have become a vital detection indicator. In our study, through the univariate Cox risk proportional regression model, total CTCs, pathology, and CTC-WBC clusters were all independent prognostic factors, associated with a poor outcome, that can be used for the evaluation of metastasis-free survival. Through the best subset selection function, the optimal multifactor Cox risk proportional regression model index is obtained. At the same time, it was confirmed that total CTCs, pathology, and CTC-WBC clusters were equally meaningful in the multifactor Cox risk proportional regression model, and the effect of the model was tested. This initially suggests that the prognostic value of total CTCs, pathology type, and CTC-WBC clusters in RCC patients are important. Among them, the presence of CTC-WBC clusters, as a critical prognostic factor, suggest that the patient may have a poor outcome.

In agreement with our results, Lisa Rydén’s research shows that in breast cancer patients, whether PFS or OS, CTC-WBC clusters are associated with a poor prognosis [28]. Szczerba detected CTCs in blood samples from 70 patients with breast cancer. Most of the CTCs were single CTCs, and a few CTC clusters (8.6%) were CTC-WBC clusters (3.4%). Studies have confirmed that when compared with a single CTC or a CTC cluster, the existence of a CTC-WBC cluster is associated with a poor prognosis in patients with breast cancer [11]. In hepatocellular carcinoma, Pan’s research shows that CTC-WBC clusters in the peripheral blood are an independent predictor of DFS and OS. Their presence indicates a poor prognosis in patients with hepatocellular carcinoma [12]. Shou confirmed, by studying immunofluorescence, that neutrophil NETs could capture tumour cells. The analysis of 533 RCC samples from The Cancer Genome Atlas (TCGA) showed that the NET score is an independent prognostic factor for RCC patients, which to a certain extent illustrates that CTC-WBC clusters can be used as a poor prognostic indicator for RCC patients [29]. In the OS correlation analysis between the survival and death groups, although there were total CTCs, solid tumour diameter could also be used as an independent and combined (with CTCs) prognostic factor, and the results were statistically significant. However, CTC-WBC clusters were not statistically significant, although they can be used as a poor prognostic factor. We consider that this is due to the insufficient number of cancer deaths in this study. In addition, the study here has several limitations, such as being from a single centre and the small number of cases included in our study. In future studies, we will develop more detailed and accurate monitoring indicators through multiple centres and larger-scale patient groups.

Here, we proposed using CTC-WBC clusters and other indicators to judge the clinical prognosis of RCC patients through the Cox regression model, which has certain novelty and clinical practicability. This liquid biopsy method can provide patients with a more convenient and accurate disease monitoring system and a more timely adjustment of interventional protocols, which can have a positive influence in improving the prognosis of patients.

## Conclusions

The analysis of clinical sample data in patients with RCC shows that CTC-WBC clusters play an important role in monitoring the prognosis. Among them, total CTCs, pathology, and CTC-WBC clusters were combined as prognostic factors for the MFS of RCC patients. Total CTCs and solid tumour diameter can be combined as prognostic factors for the OS of RCC patients. These prognostic factors provide more convenient and accurate condition monitoring for renal cancer patients and can be used to actively improve the prognosis of patients.

## Supplementary Information

**Additional file 1 Fig. S1** CTC and CTC-WBC cluster counts of each RCC patient. **Fig. S2** Gene enrichment analysis results. A: Differentially expressed gene (DEG) GO/KEGG enrichment results of circulating tumour cells and corresponding primary tumour cells. B: GO/KEGG enrichment results of hub genes in DEG.

## Data Availability

If anyone request the data from this study, please contact Tie Chong (chongtie@126.com).
