# Supplementary material for: The prognostic value of circulating tumour cells (CTCs) and CTC white blood cell clusters in patients with renal cell carcinoma
Source: BMC Cancer. 2021 Jul 17;21:826. doi: 10.1186/s12885-021-08463-7 (PMC8285812; doi:10.1186/s12885-021-08463-7)

| **Gene** | **Sequence (5'→3')** |
| --- | --- |
| EpCAM | TGGTGCTCGTTGATGAGTCAAGCCAGCTTTGAGCAAATGA |
|  | AAAGCCCATCATTGTTCTGGCTCTCATCGCAGTCAGGATC |
|  | TCCTTGTCTGTTCTTCTGACCTCAGAGCAGGTTATTTCAG |
|  |  |
| CK8 | CGTACCTTGTCTATGAAGGAACTTGGTCTCCAGCATCTTG |
|  | CCTAAGGTTGTTGATGTAGCCTGAGGAAGTTGATCTCGTC |
|  | CAGATGTGTCCGAGATCTGGTGACCTCAGCAATGATGCTG |
|  |  |
| CK18 | AGAAAGGACAGGACTCAGGCGAGTGGTGAAGCTCATGCTG |
|  | TCAGGTCCTCGATGATCTTGCAATCTGCAGAACGATGCGG |
|  | AAGTCATCAGCAGCAAGACGCTGCAGTCGTGTGATATTGG |
|  |  |
| CK19 | CTGTAGGAAGTCATGGCGAGAAGTCATCTGCAGCCAGACG |
|  | CTGTTCCGTCTCAAACTTGGTTCTTCTTCAGGTAGGCCAG |
|  | CTCAGCGTACTGATTTCCTCGTGAACCAGGCTTCAGCATC |
|  |  |
| Vimentin | GAGCGAGAGTGGCAGAGGACCTTTGTCGTTGGTTAGCTGG |
|  | CATATTGCTGACGTACGTCAGAGCGCCCCTAAGTTTTTAA |
|  | AAGATTGCAGGGTGTTTTCGGGCCAATAGTGTCTTGGTAG |
|  |  |
| Twist | ACAATGACATCTAGGTCTCCCTGGTAGAGGAAGTCGATGT |
|  | CAACTGTTCAGACTTCTATCCCTCTTGAGAATGCATGCAT |
|  | TTTCAGTGGGCTGATTGGCACTTACCATGGGTCCTCAATAA |
|  |  |
| CD45 | TCGCAATTCTTATGCGACTCTGTCATGGAGACAGTCATGT |
|  | GTATTTCCAGCTTCAACTTCCCATCAATATAGCTGGCATT |
|  | TTGTGCAGCAATGTATTTCCTACTTGAACCATCAGGCATC |

Table S1 Nucleic acid probe sequence

Figure S1


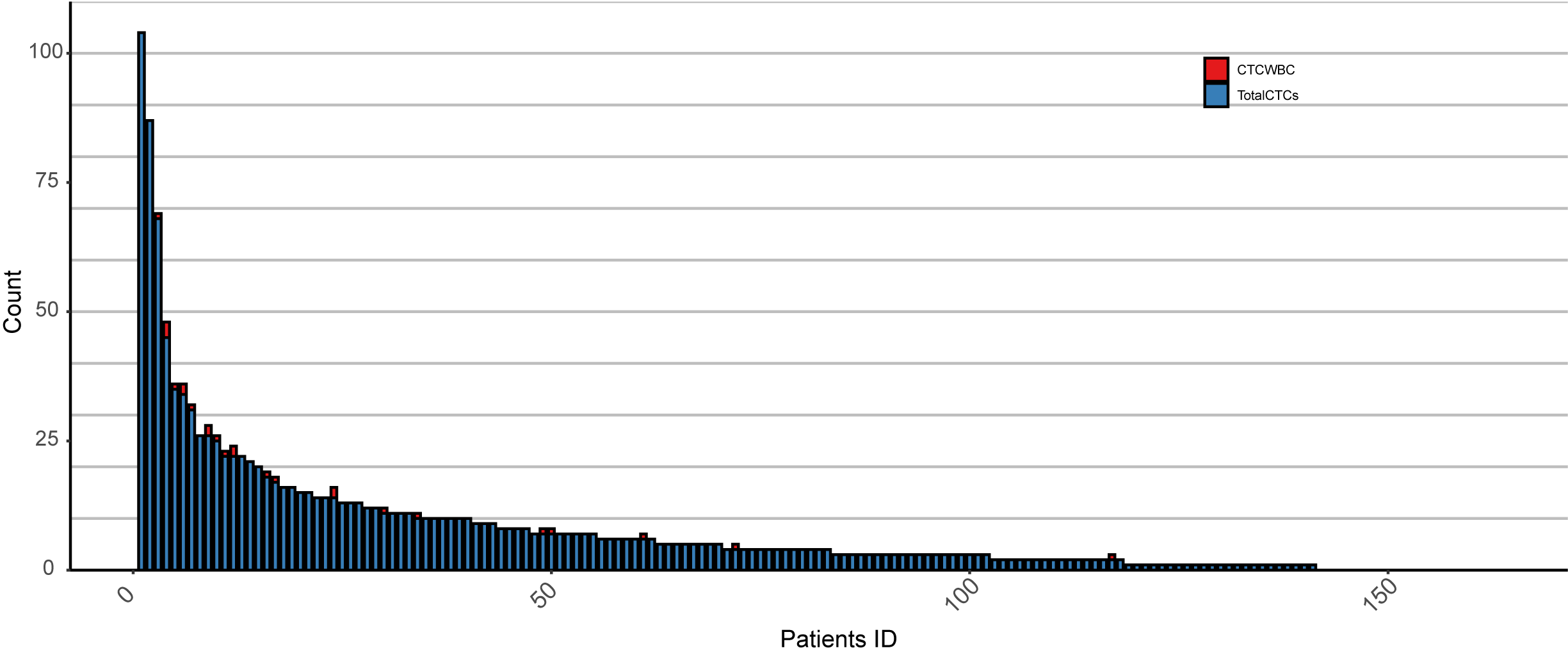


Figure S2


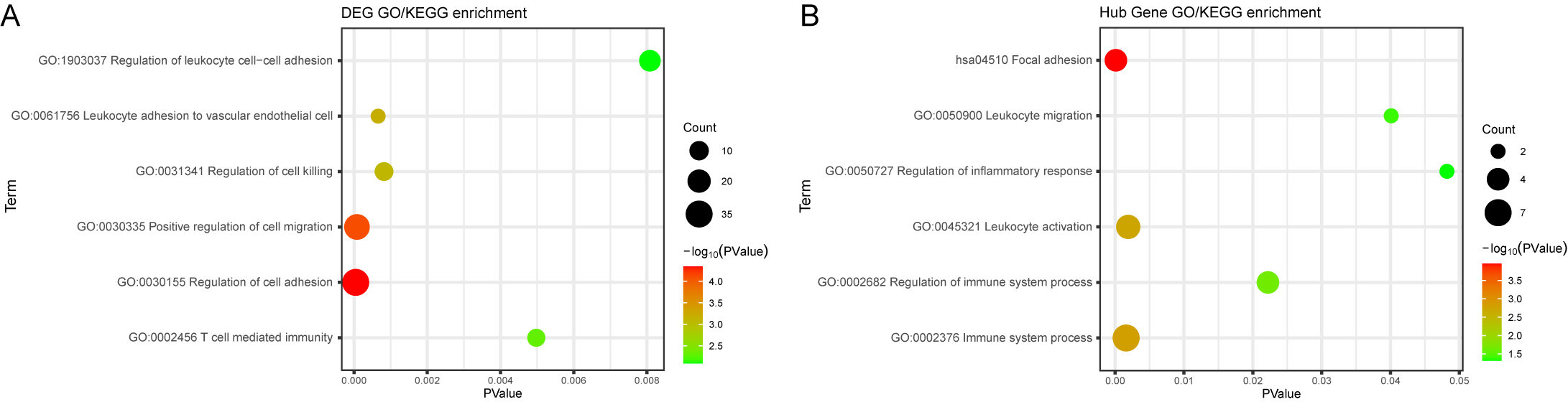

Supplement: Supplementary file 1 — Additional file 1 Fig. S1 CTC and CTC-WBC cluster counts of each RCC patient. Fig. S2 Gene enrichment analysis results. A: Differentially expressed gene (DEG) GO/KEGG enrichment results of circulating tumour cells and corresponding primary tumour cells. B: GO/KEGG enrichment results of hub genes in DEG. [file 12885_2021_8463_MOESM1_ESM.docx]
